# Electron Scattering from NO_2_: Cross Sections in the Energy Range of 1–1000 eV

**DOI:** 10.3390/molecules31010006

**Published:** 2025-12-19

**Authors:** Ana I. Lozano, Adrián García-Abenza, Jaime Rosado, Francisco Blanco, Juan C. Oller, Paulo Limão-Vieira, Gustavo García

**Affiliations:** 1 Instituto de Física Fundamental, Consejo Superior de Investigaciones Científicas, Serrano 113-bis, 28006 Madrid, Spainagarciaa@aemet.es (A.G.-A.); 2 Institute de Recherche en Astrophysique et Planétologie (IRAP), Université Toulouse III—Paul Sabatier, 9 Avenue du Colonel Roche, 31028 Toulouse, France; 3 Agencia Estatal de Meteorología (AEMET), Centro Meteorológico de Málaga, Calle Demóstenes 4, 29010 Málaga, Spain; 4 Departamento de Estructura de la Materia, Física Térmica y Electrónica eIPARCOS, Universidad Complutense de Madrid, 28040 Madrid, Spain; jaime_ros@fis.ucm.es (J.R.);; 5 División de Tecnología e Investigación Científica, Centro de Investigaciones Energéticas, Medioambientales y Tecnológicas, 28040 Madrid, Spain; jc.oller@ciemat.es; 6 Laboratório de Colisões Atómicas e Moleculares, CEFITEC, Departamento de Física, Faculdade de Ciencias e Tecnologia, Universidade NOVA de Lisboa, 2829-516 Caparica, Portugal; plimaovieira@fct.unl.pt

**Keywords:** electron collisions, electron-molecule interaction cross sections, elastic and inelastic scattering

## Abstract

Total electron scattering cross sections (TCSs) for NO_2_ molecules have been measured with a magnetically confined electron beam transmission apparatus for impact energies ranging from 1 to 200 eV. The estimated total uncertainty limits are within ±5%. Moreover, integral elastic, ionization, electronic and rotational excitation cross sections in the (20–1000 eV) energy range have been calculated with our independent atom model-based screening corrected additivity rule including interference effects (IAM-SCAR+I) method. The cross-section data set derived from this study is critically compared with the recommended values available in the literature.

## 1. Introduction

Nitrogen oxides (N_2_O, NO, NO_2_) are highly reactive chemical species which are important to understand different natural processes. In particular, nitrogen dioxide is a hazardous air pollutant, especially in urban areas [1]. It is produced by the combustion of fossil fuels and other industrial processes and can be lethal for living organisms [2]. Its main effect on human health is related to respiratory diseases, including asthma [3]. It also has atmospheric consequences by leading to acid rain that damages infrastructure and agricultural activities while triggering chemical reactions in the troposphere [4], where it is mainly generated by lightning, impacting ozone levels. Many of these processes are mediated by electrons generated by different sources, making electron interactions with these molecular species a significant area for investigation.

NO_2_ is a basic triatomic molecule which presents some similarities to CO_2_ and O_3_, and, although not so extensively as for these molecules, electron scattering from NO_2_ has motivated relatively recent theoretical investigations. Curik et al. [5], using a single-center expansion (SCE) approach, calculated the integral electron elastic cross section from NO_2_ and analyzed its low-energy single-particle resonances. O^−^ fragment generation, via dissociative electron attachment (DEA), has been detected experimentally [6,7,8,9,10,11,12]. Later on, Munjal et al. [13] used the R-matrix method to calculate elastic, electronic excitation and total electron scattering cross sections for NO_2_ for impact energies from 0 to 12 eV. Gupta et al. [14], also with the R-matrix method, found similar results and also reported total electron scattering cross sections for energies above 10 eV using a spherical model potential representation. The process of electron attachment (EA) to NO_2_ has theoretically been investigated by Liu et al. [15]. Different model potentials and independent atom formalisms were also used by Joshipura et al. [16,17], Zecca et al. [18], Shi et al. [19], Sun et al. [20] and Kumer et al. [21] to calculate intermediate and high energy electron elastic and inelastic cross sections. From the experimental side, the total electron scattering cross sections (TCSs) measured by Szmytkowski et al. [22,23] and Zecca et al. [24] are considered as the most reliable TCS values available in the literature. Ionization cross sections have also been measured [25,26,27,28] and calculated [29,30] by different authors. Electron scattering data up to 2001 were compiled by Karwasz et al. [31] and further supplemented by Song et al. in 2019 [32], who presented a complete set of recommended cross section data. In spite of these studies, important inconsistencies exist in the available cross section data.

Here, we present accurate TCS values (within ±5%) of NO_2_ for electron impact energies in the range of (1–200 eV) as measured with a magnetically confined electron beam transmission apparatus. In addition, integral elastic, electronic excitation and ionization cross sections have been calculated using our independent atom model-based screening corrected additivity rule (IAM-SCAR) method [33], including interferences (IAM-SCAR+I) [34], for electron impact energies ranging from 20 to 1000 eV.

## 2. Results

**Figure 1:**
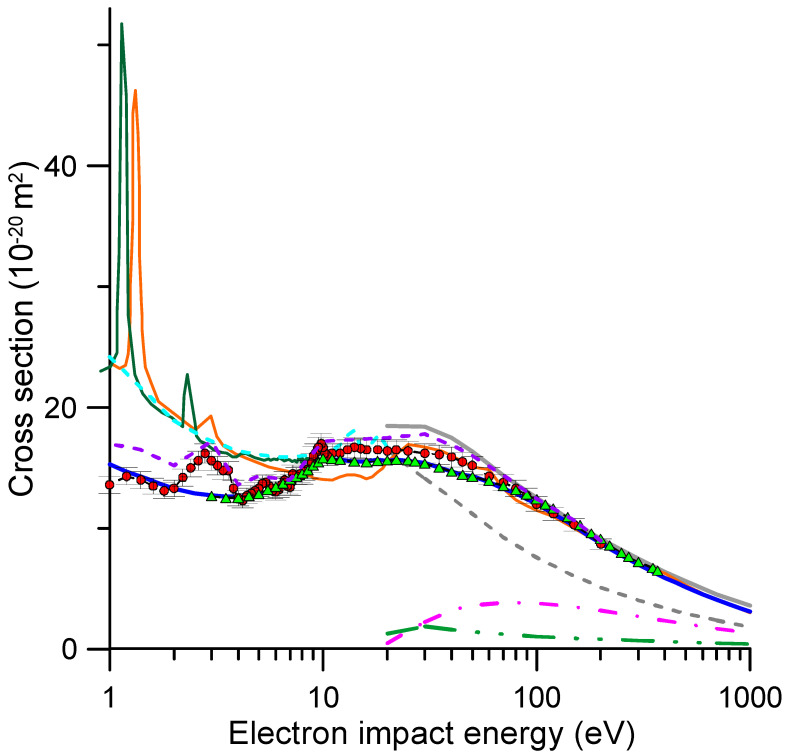
Integral electron scattering cross sections for NO_2_ molecules. ●, present experimental TCS results; **---**, present experimental TCS, including the correction for missing angles shown in Table 1; ▲, experimental TCS values from Ref. [23]; ▬, recommended TCS data from Ref. [32]; ▬, present TCS calculation; ▬, calculated integral elastic cross sections from Ref. [14]; ▬, integral elastic calculation from Ref. [13]; **---**, integral elastic calculation from Ref. [5]; **---**, present integral elastic calculation; **-.-**, present ionization cross section calculation; **-..-**, present electronic excitation cross section calculation.

The present electron scattering TCSs for NO_2_ are shown in Figure 1. The numerical values are also displayed in Table 1, covering 1 to 20 eV, and 2, ranging from 20 to 1000 eV. Other experimental and theoretical data available in the literature are also plotted in this figure for comparison. The error bars of our experimental results (±5%) correspond to the random uncertainties described in Section 3. All the experimental TCSs directly measured in a transmission-beam experiment are affected by systematic errors due to the electrons elastically or rotationally scattered in the forward direction, within the acceptance angle of the detector (missing angles) [35]. The magnitude of this uncertainty, which tends to lower the measured TCS, can only be determined by integrating calculated differential cross sections (elastic and rotational, provided that other inelastic processes are discriminated by the energy analyzer in front of the detector) over these missing angles. Under magnetic confinement conditions this effect is energy-dependent (see Equation (1) in Section 3) and tends to be more relevant for decreasing energies. All the experimental results shown in Figure 1 are uncorrected for missing angles. Table 1 shows the present missing angles from 1 to 20 eV along with their estimated contribution to the measured TCS as derived from the IAM-SCAR+I-calculated differential elastic and rotational excitation cross sections (see Equation (2)). This method is based on an independent atom representation which tends to overestimate the TCS values for energies lower than 20 eV [33,34]. We should therefore consider the magnitude of the “missing angles” correction shown in Table 1 as a maximum estimate of the impact of this systematic error.

A first look at Figure 1 seems to indicate an overall good agreement between the available experimental data, except for the prominent resonance around 2.8 eV. However, a closer inspection of the lower-energy region (1–20 eV), reveals important discrepancies between the present results and those from Ref. [23], on which the recommended values of Song et al. [32] are based. The local maxima of the TCSs that we found between 1 and 10 eV, in particular around 1.2, 2.8, 5.2 and 9.8 eV, are somehow missing in the experimental results of Szmytkowski and Mozejko [23].

**Figure 2:**
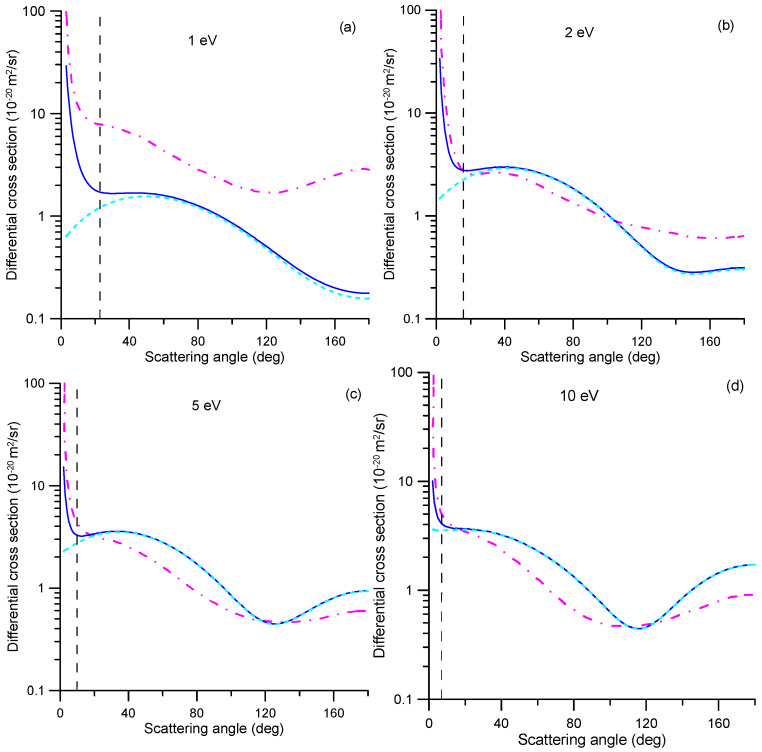
Differential electron scattering cross sections from NO_2_: ▬, IAM-SCAR+I elastic plus rotational excitation DCS; **-.-**, dipole-corrected elastic DCS from Ref. [13]; **---**, IAM-SCAR+I pure elastic DCS (without Born corrections); _ _ _, acceptance angle of the detector (missing angle). Impact electron energies: (**a**), 1 eV; (**b**), 2 eV; (**c**), 5 eV; (**d**), 10 eV.

Below 10 eV, the integral elastic cross sections (ICSs) calculated in Refs. [5,13,14] are significantly higher in magnitude than the current experimental TCSs. At these energies, the elastic ICSs should be close to the TCSs but lower in magnitude. This discrepancy is likely driven by the dipole interactions, which may be prevalent at low electron energies. As explained in Section 3.1, our experimental TCS values do not include contributions from rotational excitations, whereas these calculations incorporate dipole-Born corrections to account for such processes, which become increasingly important at lower energies. Adding our calculated correction for missing angles to the experimental TCS (see Figure 1), we obtain TCS values in reasonable agreement (within 20%) with the R-matrix calculations from Refs. [13,14], except for impact energies lower than 2 eV, where the latter can be higher by approximately 40% at 1 eV. The origin of this discrepancy is not clear, but it is important to know that the dipole-corrected ICS values from Refs. [13,14] were obtained by integrating the DCS cross section determined through the POLYDCS program [35], which relies on the Born approximation (see Ref. [13] for details). As the contributions of the missing angles have been determined by integrating our IAM-SCAR+I differential cross sections (DCS) across the detector’s acceptance angles, the corresponding DCS values at 1, 2, 5 and 10 eV are shown in Figure 2, alongside the data from Ref. [13] for comparison. At these impact energies, the magnitude of the missing angles is represented by a black dashed line. As shown in this figure, except for the impact energy of 1 eV, there is a reasonable qualitative agreement between our elastic plus rotational excitation DCS values and those dipole-Born-corrected from Ref. [13] for scattering angles greater than the detector’s acceptance angle. However, below these angles, the DCSs from Ref. [13] rise significantly quicker than ours, which would increase the magnitude of the missing angle correction. At 1 eV, in addition to this effect, the DCS values from Ref. [13] are much higher than ours by a factor of 2–4, probably due to the proximity of the resonance at 1.18 eV, which is not included in our calculation.

As Figure 2 shows, in both cases, the missing angle contributions are dominated by the Born corrections. However, the DCSs calculated in Ref. [13] are obtained by adding all rotational cross sections from the ground rotational state (*J* = 0) to all possible excited states (*J′* = 1,2,3,4). Because NO_2_ is a weakly polar molecule, the main contribution to the rotational DCS arises from the transition 0 → 1. Our approach is different [36]. Since the experiment takes place at room temperature, the initial state of the target is not *J* = 0; therefore, we assume that the initial *J* state of the molecule could be any, provided that their populations are thermically distributed at that temperature (300 K). We factor in all possible transitions *J* → *J′* = *J* ± 1 while considering an averaged effective rotational excitation energy that relies on the rotational structure of the target and the temperature (0.00214 eV at 300 K, in this case). This fact could justify an overestimation of the computed R-matrix cross section when the dipole correction is included. Unfortunately, this cannot be experimentally validated since the necessary energy resolution is not attainable, and electrons rotationally scattered within the detector’s acceptance angle will be considered unscattered.

Another relevant discrepancy relates to the position and magnitude of the calculated electron attachment resonances. Although EA processes are not elastic, they can appear as resonances in the elastic cross section calculations as consequence of the electron trapping by the potential well formed by the molecular potential field and the repulsive centrifugal barrier of the incoming electron. Some of these references were identified by Curik et al. [5] through a molecular potential analysis, although, as shown in Figure 1, these are not manifested in their elastic scattering calculation based on a single-center expansion. Figure 1 also shows that both R-matrix calculations [13,14] found two low-energy shape resonances, although those of Munjal et al. [13] are slightly shifted to lower energies. The position of the resonances from Ref. [14] at 1.3 and 3.0 eV, respectively, are in good agreement with that of the present study (1.2 and 2.8 eV) if we consider our experimental uncertainty (±0.1 eV), which is a combination of the energy resolution and the energy gap between experimental points. This uncertainty also affects the magnitude of the cross section. While, for the broad resonance around 2.8 eV, the magnitude of the calculated and experimental results are similar, the experimental value for the much narrower resonance at around 1.2 eV is noticeably reduced. This can also be affected by a reduction in transmission efficiency at these low energies (see Section 3). The positions of the resonances that we detected in the TCSs at 1.2, 2.8, 5.2 and 9.8 eV have also been identified in dissociative electron attachment (DEA) experiments [6,7,8,9,10,11,12] as O^-^ generation processes. Note that these experiments focus on detecting anion fragments after the electron attachment. A proper correlation between the present EA cross section and their DEA cross sections would require, after the formation of the molecular anion, a dynamical study of all the possible decay mechanisms, including autodetachment (leaving the neutral target molecule either vibrationally excited or not), anionic fragmentation and neutral dissociation. Such a study is out of the scope of this work but will be the subject of further investigations. Above 10 eV, our TCS values tend to be higher in magnitude than those of Refs. [23,32], but the discrepancies are within the combined uncertainty limits.

For impact energies higher than 20 eV, our experimental and theoretical TCSs agree within the uncertainty limits (maximum discrepancy of 13% at 20 eV), particularly when considering the influence of the missing angles. As shown in Table 2, subtracting the effect of the missing angles from the IAM-SCAR+I calculation (TCS-MA column), we obtain values in excellent agreement (within 6%) with the present experimental data.

Table 2 also shows our calculated integral elastic, electronic excitation and ionization cross sections for electron impact energies above 20 eV, where our IAM-SCAR+I method applies, up to 1000 eV. The TCS values calculated while considering the missing angle effect are also provided.

**Figure 3:**
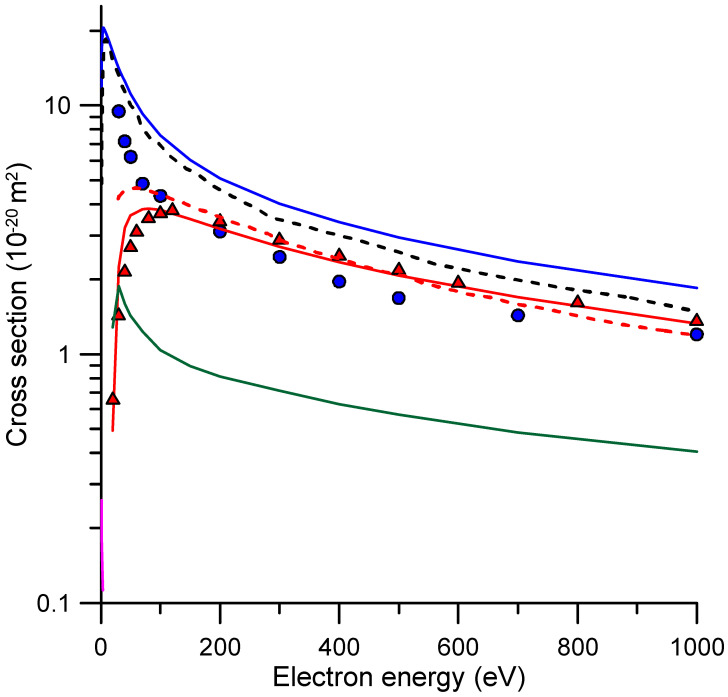
Integral electron scattering from NO_2_ cross sections. Present elastic (▬), ionization (▬), electronic (▬) and rotational (▬) excitation cross sections. Recommended elastic (●) cross sections from Song et al. [32]. Elastic (**---**) and ionization (**---**) cross sections from Kumer et al. [21]. Experimental ionization (▲) cross sections from Lindsay et al. [28].

Integral elastic, ionization, electronic excitation and rotational excitation cross sections from 20 to 1000 eV impact energies are also plotted in Figure 3. With respect to the elastic processes, the present integral cross sections (ICSs) (see Figure 3 and Table 2) are about 45–90% higher in magnitude than those recommended in Ref. [32]. In their compilation, Song et al. [32] noted the absence of experimental elastic cross section data for NO_2_ and pointed out inconsistencies in previous calculations when compared to Szmytkowski’s TCS data, which they considered the most reliable. As a result, they recommend the elastic ICSs calculated by Joshipura et al. [17]. However, as shown in Figure 3, recent calculations from Kumer et al. [21] using a screening-adjusted independent atom model agree with the present ones within the estimated uncertainty limits (±10%).

As far as ionization cross sections are concerned, we found a fairly good agreement with the recommended data of Song et al. [32], which are mainly based on the experimental values of Lindsay et al. [28], except for the position of the maximum values. In both cases, the maximum cross sections are near 100 eV, while ours reach their maximum value around 80 eV, and that of the recommended data set is located at 120 eV.

For completeness, we have included in Figure 3 and Table 2 our IAM-SCAR+I-calculated electronic excitation and the rotational excitation derived from the Born approximation [36]. At the impact energies for which this calculation is considered to be reliable within 10% (>20 eV), no previous experimental or theoretical electronic and rotational excitation cross sections have been found in the literature.

## 3. Materials and Methods

### 3.1. Experimental Methods

The magnetically confined electron beam transmission (MCEBT) apparatus used in this study to measure the total electron scattering cross sections (TCSs) of NO_2_ molecules for impact energies ranging from 1 to 200 eV has been detailed in a previous publication [36]. Thus, we only summarize here some experimental aspects which may be relevant for the discussion of the results. The entire system is surrounded by an intense (0.1 T) axial magnetic field which confines and guides the electron beam all along its constituent chambers. Briefly, the primary beam is obtained through the thermionic emission of a hairpin filament with an initial energy spread of around 500 meV. Then, it is focused into a nitrogen gas trap, where electrons are maintained around 50 ms by applying a pulsed electric potential barrier at both ends of this chamber. During this time, the primary electron beam is cooled down by successive collisions with the nitrogen molecules inside the chamber. With this procedure, as described in detail in Ref. [36], the initial energy spread of the electron beam is reduced down to around 100 meV. The electron beam is subsequently transported to the scattering chamber, where the target molecule is admitted via a leak valve. The gas pressure inside this chamber is kept constant and measured by a Baratron capacitance manometer. Transmitted electrons are energy-analyzed with a retarding potential analyzer (RPA) and finally detected by a double microchannel plate (MCP) electron multiplier operating in single counting mode. By choosing the operating point as indicated in Ref. [36], the effective energy resolution can be substantially improved, down to about 80 meV. Measurement conditions, as well as data acquisition and analysis, are controlled using custom designed LabView (National Instruments) programs.

The total electron scattering cross sections are obtained for each energy by using the well-known Beer–Lambert attenuation law and assuming an ideal gas behavior. In addition, to avoid multiple scattering processes and assure the validity of the Beer–Lambert law, a convenient range of gas pressures is determined and maintained during the attenuation measurements. To reduce statistical uncertainties to about 4%, at least five attenuation curves were measured for each impact energy. By considering all the noticed random error contributions (see Ref. [36] for details) a total uncertainty limit of ±5% is obtained for the present measurements. Basically, this total ±5% assigned to the absolute TCS values is the result of adding in quadrature the statistical uncertainties (4%), absolute pressure and temperature determination (1%) and fitting procedures (1%). At the lowest energies, around 1 eV, the effects of transmission loss might result in a reduction in the measured TCS values [36].

An important limitation of the accuracy of transmission-beam experiments is the acceptance angle (angular resolution) of the detector. Electrons elastically or rotationally scattered into this angle are not recognized as scattered electrons by the detector (“missing angle” effect [37]), resulting in a lower measured TCS value. Due to the magnetic confinement, in this experimental setup the angular resolution (Δ*θ*) is linked to the energy resolution (Δ*E*) and the incident electron energy (*E*) as expressed in the following equation:

(1) $$\Delta \theta =\;arccos\sqrt{1-(\frac{\Delta E}{E})}$$

(see Ref. [36] for further details).

In these conditions, the “missing angle” (Δ*θ*) contribution increases for decreasing energies. This tends to lower the measured TCS from the actual value, especially for lower collision energies. In addition, for polar molecules, as is the case for NO_2_, this effect will increase, since rotational excitations are strongly peaked in the forward direction and especially at the lower energies. The present results are not corrected for this systematic error. Consequently, in order to perform a fair comparison with other experimental and/or theoretical data, the experimental angular acceptance should be considered. These considerations are especially critical when we discuss rotational excitation processes. Transmission experiments with energy resolution of the order of the present one are not able to account for rotational excitations (the average rotational excitation energy of NO_2_ at 300 K is about 2.1 meV), while some calculations indirectly include rotational excitation via dipole-Born corrections [37]. However, the present energy resolution was good enough to distinguish the local maxima in the low energy TCSs that correspond to resonant electron attachment processes. In order to facilitate a further comparison of the current experimental results with other theoretical or experimental data, we have estimated the contribution of the missing angles to the measured cross sections ($\sigma \left(\theta \right)$) by employing the differential elastic and rotational cross sections (*d*(*σ_el_ + σ_rot_*)*/dΩ*) calculated with our IAM-SCAR+I (see Section 3.2) according to the following expression:

(2) $$\sigma \left(\Delta \theta \right)=2\pi (\int_{0}^{\Delta \theta }\frac{d(\sigma_{el}+\sigma_{rot})}{d\Omega }\sin\theta \;d\theta +\int_{180-\Delta \theta }^{180}\frac{d(\sigma_{el}+\sigma_{rot})}{d\Omega }\sin\theta \;d\theta ),$$

where the second integrand represents the backward scattered electrons that are redirected to the scattering chamber (see Ref. [36] for further information).

### 3.2. Theoretical Methods

Differential and integral elastic as well as integral inelastic electronic excitation and ionization cross sections have been obtained through our IAM-SCAR+I method. The calculation procedure has been described in detail in previous publications [33,34]. In summary, the molecular target is considered as an aggregate of its individual atoms, where each atom is represented by an ab initio optical potential. The real part of the potential accounts for elastic scattering, while the imaginary part represents the inelastic processes, i.e., the “absorption part”. The differential scattering cross sections are obtained from the atomic data using the screening corrected additivity rule (SCAR) procedure, incorporating interference (I) corrections by summing all the atomic amplitudes, where the phase coefficients are included. Then, by integrating over all the scattered angular ranges, the integral scattering cross sections (ICSs) are obtained. NO_2_ possesses a moderate permanent dipole moment. Although some discrepancies about its value can be found in the literature, we adopted the 0.316 D value according to Hodgeson et al.’s [38] measurements. Using this value, the rotational excitation cross sections have been separately calculated by means of the first-Born approximation. Finally, the calculated TCS is obtained by summing up the elastic, rotational excitation, electronic excitation and ionization cross sections. The uncertainties assigned to the present calculated data have been derived from comparisons with accurate experimental values. Probably the most representative molecular targets for this comparison are H_2_O and tetrahydrofurane (THF). For these molecules, we have measured, calculated and compared with available data to establish complete cross section sets [39], which have been validated through electron transport simulations [40]. We can then safely assign to the present elastic, electronic excitation, ionization and total electron scattering cross sections an overall uncertainty limit of ±10% for electron impact energies ranging from 20 to 1000 eV. Depending on the target, the IAM-SCAR+I calculation could provide a good estimation of the cross sections below 20 eV. However, in order to constrain the uncertainty within 10%, results below this limit are not included in this study.

## 4. Conclusions

In this study, we report on new experimental results of the total electron scattering cross sections of NO_2_ for impact energies ranging from 1 to 200 eV, as measured with a magnetically confined electron beam transmission apparatus. The reduced uncertainty limits (±5%) and effective energy resolution (80 meV) of the present experimental conditions enabled the detection of some local maxima in the TCSs that had previously gone unobserved. Integral elastic, ionization, electronic and rotational excitation cross sections have been calculated using our IAM-SCAR+I method for impact energies from 20 to 1000 eV. For this impact energy range, this calculation procedure is proven to be accurate within 10%. The present elastic cross sections show a good agreement with the recent model potential calculations of Ref. [20]. However, they are higher, by more than 40%, than the data recommended by Song et al. [32]. With respect to the ionization cross section, except for the position of the maximum value, our calculation agrees well with previous measurements. No previous total electronic and rotational excitation cross sections have been found in the literature, but those given by the present calculation are consistent with our measured TCSs in the overlapping energy domain (20–200 eV).

As a consequence of this research, it is clear that, for the lower impact energies (1–10 eV), further theoretical and experimental investigations are necessary to correlate the current local maxima in the experimental TCSs with temporary anion formation and their decay processes, along with a better characterization of the elastic scattering processes at intermediate and high energies (10–1000 eV). Furthermore, the recently recommended data from Ref. [32] require to be revised and updated to incorporate the new insights and cross sectional values reported in this study.

## Figures and Tables

**Table 1:** Present experimental TCS results (±5%) in units of 10^−20^ m^2^ along with the missing angles (Δ*θ*) in degrees and the estimated contribution of the elastic and rotational scattering in the missing angles to the TCS (in units of 10^−20^ m^2^), as calculated with our IAM-SCAR+I method; *σ*(Δ*θ*), in units of 10^−20^ m^2^.

E (eV)	TCS_exp_	Δ*θ*	*σ*(Δ*θ*)	E (eV)	TCS_exp_	Δ*θ*	*σ*(Δ*θ*)
1.0	13.6	22.8	3.4	8.0	14.4	7.9	
1.2	14.3	20.7		8.2	14.7	7.8	
1.4	14.4	19.1	2.5	8.5	14.9	7.6	
1.6	13.5	17.8		8.8	15.4	7.5	
1.8	13.1	16.8		9.0	16.0	7.4	
2.0	13.3	15.9	1.9	9.2	16.2	7.3	
2.2	14.2	15.1		9.5	16.7	7.2	
2.4	15.0	14.5		9.8	17.0	7.1	
2.6	15.6	13.9		10.0	16.7	7.0	0.52
2.8	16.2	13.4		10.2	16.2	6.9	
3.0	15.6	12.9	1.6	10.5	16.0	6.9	
3.2	15.2	12.5		11.0	16.2	6.7	
3.4	14.8	12.1		12.0	16.2	6.4	
3.6	14.8	11.8		13.0	16.5	6.2	
3.8	13.3	11.5		14.0	16.7	5.9	
4.0	12.5	11.2	1.1	15.0	16.6	5.7	0.77
4.2	12.3	10.9		16.0	16.5	5.6	
4.4	12.7	10.6		18.0	16.5	5.2	
4.6	12.9	10.4		20.0	16.4	5.0	1.1
4.8	13.1	10.2		22.0	16.5	5.5	
5.0	13.3	10.0	1.0	25.0	16.4	5.1	
5.2	13.7	9.8		30.0	16.2	4.7	1.6
5.4	13.8	9.6		35.0	16.1	4.3	
5.6	13.5	9.4		40.0	15.9	4.0	1.0
5.8	13.1	9.2		45.0	15.5	3.8	
6.0	13.0	9.1		50.0	15.2	3.6	0.81
6.2	13.2	8.9		60.0	14.3	3.3	
6.4	13.6	8.8		70.0	13.8	3.1	0.58
6.6	14.0	8.7		80.0	13.3	2.9	
6.8	13.5	8.5		90.0	12.7	2.7	
7.0	13.4	8.4	0.6	100	12.0	2.6	0.45
7.1	13.8	8.3		120	11.2	2.3	
7.2	14.5	8.3		150	10.3	2.1	0.34
7.5	14.3	8.1		200	8.7	1.8	0.27
7.8	14.5	8.0					

**Table 2:** Elastic, ionization, electronic excitation (EE), rotational excitation (RE) and total electron scattering cross sections (TCSs) calculated with our IAM-SCAR+I method. The TCS values calculated while considering the missing angle effect are also provided (TCS-MA). All cross sections in units of 10^−20^ m^2^.

E (eV)	Elastic	Ionization	EE	RE	TCS	TCS-MA
20	16.5	0.49	1.28	0.26	18.5	17.4
30	14.1	2.24	1.88	0.18	18.4	16.8
40	12.5	3.22	1.60	0.14	17.5	16.4
50	11.2	3.61	1.43	0.11	16.3	15.6
70	9.24	3.84	1.23	0.08	14.39	13.8
100	7.59	3.81	1.04	0.06	12.5	12.1
150	6.05	3.50	0.90	0.04	10.5	10.2
200	5.10	3.19	0.81	0.03	9.1	8.8
300	4.03	2.70	0.71	0.02	7.5	7.3
400	3.39	2.34	0.63	0.02	6.4	6.2
500	2.94	2.07	0.57	0.01	5.6	5.5
700	2.36	1.69	0.48	0.01	4.5	4.4
1000	1.84	1.33	0.41	0.01	3.6	3.5

## Data Availability

All data supporting the reported results are included in the article. Further inquiries can be directed to the corresponding author.
